# Who stays? Understanding the attrition in a longitudinal aging study of older Chinese immigrants in the United States

**DOI:** 10.1093/geroni/igaf098

**Published:** 2025-09-14

**Authors:** Yanping Jiang, Yuyang Zhu, Stephanie Bergren, Wendi Da, Dexia Kong, Fengyan Tang

**Affiliations:** Institute for Health, Health Care Policy and Aging Research, Rutgers, The State University of New Jersey, New Brunswick, New Jersey, United States; Department of Family Medicine and Community Health, Rutgers, The State University of New Jersey, New Brunswick, New Jersey, United States; Institute for Health, Health Care Policy and Aging Research, Rutgers, The State University of New Jersey, New Brunswick, New Jersey, United States; Institute for Health, Health Care Policy and Aging Research, Rutgers, The State University of New Jersey, New Brunswick, New Jersey, United States; Institute for Health, Health Care Policy and Aging Research, Rutgers, The State University of New Jersey, New Brunswick, New Jersey, United States; Department of Social Work, The Chinese University of Hong Kong, Hong Kong, China; School of Social Work, University of Pittsburgh, Pittsburgh, Pennsylvania, United States

**Keywords:** Participation attrition, Aging research, Older immigrants, Attrition patterns

## Abstract

**Background and Objectives:**

Despite the rapid growth of older Asian American populations, little is known about their retention and its associated factors in longitudinal aging studies, partially due to the limited longitudinal studies among this population. This study addresses this critical gap by examining key predictors of attrition in this understudied population.

**Research Design and Methods:**

Using data from the Population Study of Chinese Elderly in Chicago (PINE), a large longitudinal epidemiological study of older Chinese immigrants in the United States (US), we analyzed the effect of sociodemographic, immigration, health, and psychosocial characteristics on attrition status and various attrition patterns.

**Results:**

High levels of acculturation and longer length of stay in the US were associated with a higher likelihood of attrition. Also, participants with higher levels of educational attainment and loneliness were more likely to drop out of the study earlier. In addition, participants with lower income were more likely to remain in the study.

**Discussion and Implications:**

These results show unique attrition dynamics in older Asian immigrants, where acculturation and education paradoxically increase attrition risk. These findings highlight the need for tailored retention strategies to enhance continued participation in longitudinal aging studies among older Asian American immigrants.

Innovation and Translational Significance This study, for the first time, identifies targets to enhance research retention among older Asian Americans—a rapidly growing yet underrepresented population in aging studies. We examined the effect of various sociodemographic, immigration, health, and psychosocial factors on attrition in a large longitudinal study of older Chinese immigrants. By showing the unique effects of acculturation, loneliness, and socioeconomic factors on attrition, our findings provide a roadmap for developing culturally relevant strategies to enhance retention in aging cohort studies.

Successful recruitment and retention of racial and ethnic minority populations in aging research is critical to advancing the understanding of healthy aging and health disparities. However, certain populations have been historically excluded from aging research, including Asian Americans (Kelley & Thorpe Jr, 2023), despite Asian Americans being one of the ­fastest-growing racial and ethnic minority populations in the United States (US). (Budiman et al., 2019). The lack of representation of Asian Americans in aging studies is partly due to recruitment challenges, often related to language barriers, research mistrust, and immigration status (George et al., 2014; Katigbak et al., 2016). Although there is a growing research effort to identify barriers to recruiting Asian Americans for health research, little is known about the factors affecting attrition among Asian Americans who have already participated in research. This knowledge gap is critical as the scientific community increasingly emphasizes the need for greater representation of Asian Americans in aging health research. Understanding factors contributing to attrition in this population is essential for developing tailored strategies to enhance retention in future longitudinal aging studies and ensuring the representativeness and validity of findings.

The aim of this study was to examine the factors related to participant attrition in the Population Study of Chinese Elderly in Chicago (i.e., PINE; Dong et al., 2014). To our knowledge, the PINE study is the largest longitudinal epidemiological aging study of older Chinese immigrants in the US. The PINE study was assembled between 2011 and 2013 and consisted of 3,157 Chinese aged 60 years and older living in the Greater Chicago area. Previous studies have examined the representativeness of the PINE sample (Simon et al., 2014). However, limited work has been done to understand panel attrition, which could undermine the representativeness of the sample over time. Importantly, findings from such an investigation can offer valuable insights for improving research retention in future longitudinal aging studies targeting older Asian immigrant populations.

Panel attrition, the loss of participants enrolled at the baseline during follow-ups throughout longitudinal studies (de Leeuw & Lugtig, 2014), can reduce the validity and generalizability of a study's findings (Barry, 2005). Participants who remain in the study may differ systematically from those who drop out and, therefore, are no longer representative of the target population. Furthermore, high attrition rates can reduce sample size and the statistical power to get robust population estimates. In aging cohorts, as participants staying in the study tend to have systematically better physical health and cognitive function than those dropping out, severe positive bias caused by attrition was detected (Caracciolo et al., 2008; Muszyńska-Spielauer & Spielauer, 2022).

Previous studies have examined factors associated with an increased likelihood of attrition in longitudinal aging cohorts. For example, sociodemographic factors, including older age, living alone and not being married, and lower income and educational attainment, have been associated with an increased risk of attrition (Cacioppo & Cacioppo, 2018; Kuh et al., 2016). Health factors such as poor cognitive functioning, self-rated health, and medical comorbidities were also found to predict attrition in aging cohorts (Burke et al., 2019; Cacioppo & Cacioppo, 2018; Chang et al., 2009; Kuh et al., 2016; Salthouse, 2014). In addition, psychological distress, particularly depressive symptoms, has been linked to increased dropout rates (Burke et al., 2019; Cacioppo & Cacioppo, 2018; Chang et al., 2009; Mein et al., 2012). However, few studies have examined the effect of other psychological distress, such as social isolation and loneliness, on attrition in aging cohorts. Furthermore, whether immigration-related factors, such as acculturation, may influence participation retention has been rarely examined.

Immigration-related factors may influence retention in longitudinal studies of aging immigrant populations. For example, longer length of stay in the host country and low acculturation have been linked to two key risk factors for attrition: older age and lower cognitive function, respectively (Mendoza et al., 2022; Xu et al., 2017). Although no studies, to our knowledge, have explicitly examined the effect of these immigration-related factors on attrition, a small body of literature has documented their potential effects on research participation (Li et al., 2016). For example, a prior study indicated that longer length of stay in the US was linked to a higher likelihood of refusing research participation among older Asian immigrants (Brugge et al., 2005). Furthermore, a review study found that limited English proficiency, low acculturation, and longer length of stay in the US were significant barriers to recruiting older Chinese immigrants for clinical trials (Li et al., 2016). These findings highlight the importance of examining immigration-related factors as predictors of attrition in longitudinal aging studies, providing a foundation for developing tailored strategies to address barriers to participant retention among older immigrant populations.

In addition, to identify key predictors of attrition and develop strategies to minimize bias, it is crucial to understand potential heterogeneous patterns of attrition in longitudinal studies. Attrition is not uniform. Participants may drop out of longitudinal studies at different waves (e.g., early *vs* late drop-out) or intermittently return, and each attrition pattern may have distinct implications for tailoring retention strategies. Previous research has largely focused on monotone attrition, where participants either participated in every eligible wave in the panel or dropped out permanently, overlooking the complexities of attrition patterns (e.g., early *vs* late drop-out; Lugtig, 2014). Examining predictors of distinct attrition patterns may elucidate participant characteristics associated with early drop-out, intermittent participation, and eventually late drop-out after participating in earlier assessments. Findings from such an examination can inform tailored retention strategies (e.g., early retention interventions for early drop-out, re-engagement efforts for intermittent participation) aimed at enhancing continued participation, thereby mitigating estimation bias associated with attrition in longitudinal studies. Understanding factors contributing to various attrition patterns is particularly salient in aging cohort studies because many existing studies, such as the Health Retirement Study (HRS) and Midlife in the United States Study (MIDUS), were designed with an attempt to bring temporary drop-out back into the study in subsequent waves (Song et al., 2021; Kapteyn et al., 2006).

To fill the critical scientific gap in understanding participation attrition among older Asian immigrant populations and the complexities of attrition patterns, this study aimed to examine attrition patterns and associated factors in a large epidemiological cohort study of older Chinese adults in the US. The following research questions are addressed: (1) What are the observed attrition patterns in the PINE study? (2) How do participants who completed all follow-ups differ from those who followed other attrition patterns?

## Methods

### Participants

This study used data from the PINE study, which was first launched in 2011–2013 (Dong et al., 2014). At baseline (Wave 1), a total of 3,157 adults aged 60 years or older (58% female, mean age = 72.8 years) who self-identified as Chinese were enrolled and completed the assessment. Recruitment was conducted through collaborations with over 20 social services agencies, community centers, and organizations in the Greater Chicago area. Trained bilingual/multilingual research staff administered the survey via face-to-face in-home interviews with participants in their preferred language or dialect (e.g., Mandarin, Cantonese). Follow-ups were conducted every two years after the initial interview during 2013–2015 (Wave 2), 2015–2017 (Wave 3), 2017–2019 (Wave 4), and 2019–2020 (Wave 5), respectively.

Several contact and engagement strategies were used to maximize retention. At the end of each interview, research staff gathered additional contact information for each participant's family and friends in case they could not be reached through the primary contact. During each wave of the study, research staff would attempt to call participants and, if necessary, additional contact persons via phone. Depending on the reason for the lack of contact (i.e., disconnected number, wrong phone number, etc.), participants would be mailed a self-addressed stamped envelope to send the research team the correct information, or staff would go door-to-door and canvass non-respondents to gather their interest in continued participation. Regarding engagement strategies between waves of data collection, participants were regularly mailed tokens of appreciation for their contribution to the research. The first was a community report written in English and Chinese that described the PINE study and descriptive findings from the baseline interview. Participants were also sent annual birthday cards and calendars. Last, each subsequent interview would have an increased remuneration amount to acknowledge the contribution of participants’ time. Written consent forms were obtained from all participants. The PINE study was approved by the institutional review boards at Rush University Medical Center and Rutgers University.

### Measures

#### Attrition

Attrition status (dependent variable) was first coded as a binary variable (0 = retention, 1 = attrition). Specifically, among participants who were alive by Wave 5, those who completed all five waves of surveys were categorized as the retention group, and those who missed one or more follow-up assessments were categorized as the attrition group. To capture the complexity of the attrition pattern, we further categorized the attrition group into three patterns: early drop-out (i.e., permanently withdraw before Wave 3), late drop-out (i.e., permanently withdraw at Wave 3 or later waves), and drop-in (i.e., intermittent participation), following attrition patterns indicated in previous studies (Lugtig, 2014).

#### Mortality status

Participants’ vital status was tracked during follow-up contacts via family, social contacts, or obituary searching. By the end of Wave 5, 544 participants were deceased.

#### Participant characteristics at baseline

Key characteristics (independent variables) were selected based on existing literature examining participation retention in longitudinal aging studies (Bhamra et al., 2008; Davies et al., 2014; Radler & Ryff, 2010; Song et al., 2021). Sociodemographic characteristics included age, sex (0 = male, 1 = female), marital status (0 = others, 1 = married), years of education, income (1 = $0-4,999/year, 2 = $5,000-9,999/year, 3 = $10,000-14,999/year, 4 = $15,000 and above/year), and whether having health insurance (0 = no, 1 = yes). Immigration-related factors included length of stay in the US and acculturation. Acculturation was measured using a 12-item scale representative of three domains (i.e., language use, media use, and social relations) on a five-point scale (1 = only Chinese/all Chinese, 5 = only English/All Americans), which was adapted from the Short Acculturation Scale for Hispanics (Marin et al., 1987). A composite acculturation score was derived by summing responses on each item, with a higher score indicating a higher level of acculturation (Cronbach’s α = 0.91).

Health variables included self-rated overall health status (1 = very good/good, 2 = fair, 3 = poor), total number of self-­reported chronic medical conditions (i.e., heart disease, stroke or brain hemorrhage, cancers, high cholesterol, diabetes, high blood pressure, broken/fractured hip, thyroid disease, or osteoarthritis/joint issues), depressive symptoms, and cognitive function. Specifically, depressive symptoms were measured with the nine-item Patient Health Questionnaire (PHQ-9; Chen et al., 2013; Spitzer et al., 1999) on a four-point scale assessing the severity of each depressive symptom (from 0 = not at all to 3 = nearly every day). A sum score was created for depressive symptoms, with a higher score indicating more depressive symptoms (Cronbach’s α = 0.81). Cognitive function was assessed with a battery of cognitive tests, including the 11-item Symbol Digit Modalities Test for executive function (Smith, 1973), the East Boston immediate and delayed recall test for episodic memory (EBMT; Albert et al., 1991), the Digit Span Backwards drawn from the Wechsler Memory Scale-Revised for working memory (Wechsler, 1987), and the Chinese version of the Mini-Mental State Examination for global cognition (MMSE; Folstein et al., 1975). A composite score for cognitive function was calculated by averaging standardized scores from each of the five tests, and a higher score indicated better cognitive function (Cronbach’s α = 0.88).

Social well-being variables included perceived social support, social strain, social engagement, and loneliness. Perceived social support was measured with six items asking how often participants felt supported by their spouse/partner, family members, and friends, with response options ranging from 1 = hardly ever to 3 = often (Cornwell & Waite, 2009). A sum score was created for perceived social support, with a higher score indicating more perceived social support (Cronbach’s α = 0.69). Social strain was evaluated using six items on a three-point scale (1 = hardly ever, 3 = often), assessing how often participants receive too many demands and criticism from spouse/partner, family members, and friends (Cornwell & Waite, 2009). A composite score for social strain was calculated by summing responses on the six items, with a higher score indicating more perceived strain (Cronbach’s α = 0.60). Social engagement was assessed using a 16-item scale (Zhang et al., 2018) measuring the frequency of cognitive (e.g., watching TV, reading newspapers) and social activities (e.g., going out for activities, visiting community centers). Response options varied from 0 = once a year or less/never to 4 = every day or almost every day/20 or more times for all questions, except for the question, “How much time do you spend reading each day?,” for which the responses ranged from 0 = none to 4 = more than 3 hr. A sum score for social engagement was calculated, with a higher score indicating a higher level of social engagement (Cronbach’s α = 0.75). Loneliness was assessed using the UCLA three-item loneliness scale that asked how often participants felt a lack of companionship, left out, and isolated, with response options ranging from 0 = hardly ever to 2 = often (Hughes et al., 2004). A sum score was calculated for loneliness, and a higher score represented a higher level of loneliness (Cronbach’s α = 0.78).

### Statistical Analysis

Descriptive statistics were used to describe sociodemographic, immigration, health, and social well-being characteristics of the sample at baseline (Wave 1). Chi-square or analysis of variance tests with Bonferroni correction were used to compare differences in characteristics across the attrition status (retention *vs* attrition) and mortality status. Although mortality-related attrition is not the focus of this study, we included individuals who decreased during the study period in descriptive analyses to provide a complete description of the PINE participants. To examine the association between participant characteristics and attrition, binary and multinomial logistic regression models were, respectively, performed to examine the effect of participant characteristics on attrition status (retention *vs* attrition) and attrition patterns (retention *vs* early drop-out *vs* late drop-out *vs* drop-in). All analyses were performed using SAS version 9.4 (SAS Institute, Cary, NC). All tests were two-sided, with a *p*-value < .05 considered statistically significant.

## Results

Table 1 displays the number of participants who did not complete the survey assessment at each wave. Specifically, among the participants who were alive, the proportions who did not complete the assessment at each follow-up were 9.7%, 15.6%, 17.1%, and 17.6% at Waves 2 to 5, respectively. Table 2 displays the number of participants in each of the observed attrition pattern groups. Among 2,613 participants who were alive by Wave 5, 1,938 (74.2%) completed all assessments (i.e., the retention group), and 675 (25.8%) missed at least one ­follow-up assessment (i.e., the attrition group). Among participants in the attrition group, 287 (42.5%) were categorized as early drop-out, 129 (19.1%) were late drop-out, and 259 (38.4%) were drop-in.

**Table 1.** Cross-wave completion.

Completion status	Wave 1	Wave 2	Wave 3	Wave 4	Wave 5
	2011– 2013	2013– 2015	2015– 2017	2017– 2019	2019– 2020
**Interviewed**	3,157	2,713	2,373	2,228	2,154
**Deceased**	0	153	194	121	76
**Attrition**	0	291	437	461	459

**Table 2.** Attrition patterns among the PINE cohort who were alive by Wave 5 (*N *= 2,613).

Attrition pattern	Wave 1	Wave 2	Wave 3	Wave 4	Wave 5	*N*
**Retention**	X	X	X	X	X	1,938
**Attrition**						
** Early drop-out (*n *= 287)**	X	O	O	O	O	169
	X	X	O	O	O	118
** Late drop-out (*n *= 129)**	X	X	X	O	O	62
	X	X	X	X	O	67
** Drop-in (*n *= 259)**	X	O	X	X	X	42
	X	O	O	X	X	18
	X	O	O	O	X	24
	X	X	O	X	O	13
	X	X	O	X	X	61
	X	X	O	O	X	21
	X	O	X	O	X	6
	X	O	X	O	O	17
	X	O	O	X	O	7
	X	X	X	O	X	44
	X	O	X	X	O	6

*Note*. X = interviewed; O = did not interview.

Table 3 displays sample characteristics by attrition status and mortality status. Compared with the retention group, the attrition group had higher levels of acculturation, had longer length of stay in the US, and reported higher levels of loneliness but lower levels of perceived social support. Not surprisingly, those who deceased were the oldest and reported the worst health outcomes, including overall health status, medical comorbidities, depressive symptoms, and global cognition, compared with the retention and attrition groups, who remained alive throughout the study period.

**Table 3.** Sample characteristics by attrition and mortality status.

Variables	Overall (*N *= 3,157)	Retention^a^ (*n *= 1,938)	Attrition (*n *= 675)	Deceased (*n *= 544)	*p*
**Age**	72.8 (8.3)	71.2 (7.4)	71.4 (7.7)	**80.5 (7.9)**	<.001
**Female (*n*, %)**	1,829 (57.9)	1,170 (60.4)	403 (59.7)	**256 (47.1)**	<.001
**Married (*n*, %)**	2236 (71.0)	1,435 (74.1)	477 (71.2)	**324 (59.6)**	<.001
**Education**	8.7 (5.1)	8.9 (5.0)	9.3 (5.1)	**7.4 (5.0)**	<.001
**Acculturation**	15.3 (5.1)	15.0 (4.2)	**16.5 (7.1)**	14.5 (4.8)	<.001
**Length of stay in the United States**	20.0 (13.2)	18.2 (11.5)	**20.7 (15.6)**	**25.8 (13.9)**	<.001
**Health insured (*n*, %)**	2,385 (76.0)	1,413 (73.3)	469 (70.0)	**503 (93.0)**	<.001
**Annual income (*n*, %)**					<.001
** $0-$4,999**	1,040 (33.3)	669 (34.8)	**246 (36.9)**	**125 (23.5)**	
** $5,000-$9,999**	1,616 (51.8)	965 (50.2)	**298 (44.7)**	**353 (66.5)**	
** $10,000-$14,999**	310 (9.9)	203 (10.6)	**68 (10.1)**	**40 (7.5)**	
** $15,000 and over**	155 (5.0)	87 (4.5)	**55 (8.3)**	**13 (2.5)**	
**Overall health status (*n*, %)**					<.001
** Very good/Good**	1,236 (39.2)	770 (39.7)	282 (41.8)	**184 (33.8)**	
** Fair**	1,320 (41.8)	833 (43.0)	280 (41.5)	**207 (38.1)**	
** Poor**	601 (19.0)	335 (17.3)	113 (16.7)	**153 (28.1)**	
**Medical comorbidities**	2.1 (1.5)	2.0 (1.4)	2.0 (1.4)	**2.4 (1.6)**	<.001
**Depressive symptoms**	2.7 (4.1)	2.5 (4.0)	2.5 (4.0)	**3.3 (4.6)**	.001
**Loneliness**	0.6 (1.2)	0.5 (1.1)	**0.7 (1.3)**	**0.8 (1.4)**	<.001
**Cognitive function**	−0.04 (0.82)	0.06 (0.75)	0.06 (0.80)	**−0.53 (0.94)**	<.001
**Perceived social support**	12.7 (3.1)	13.0 (3.0)	**12.7 (3.2)**	**11.7 (3.1)**	<.001
**Social strain**	6.7 (1.2)	6.7 (1.3)	6.7 (1.1)	6.5 (1.0)	<.001
**Social engagement**	20.8 (9.2)	21.5 (9.0)	21.4 (8.8)	17.4 (9.5)	<.001

*Note*. Values in boldface indicate significant differences from the retention group based on the Chi-square or ANOVA test with Bonferroni correction. Numbers did not sum to the total sample size for some variables due to missing data.

a Reference group.

Table 4 displays the effect of participant characteristics on attrition status (i.e., retention *vs* attrition). It is of note that the sample was 2,541 in the regression analyses, due to that a small percentage (2.8%) of participants who had missing data on predictors were dropped from the regression analyses. Results showed that higher acculturation and longer length of stay in the US were associated with a higher risk of attrition (OR = 1.04, 95% CI = [1.021, 1.064]; OR = 1.01, 95% CI = [1.005, 1.022], respectively). Also, having health insurance was associated with a decreased risk of attrition (OR = 0.70, 95% CI = [0.546, 0.896]). In addition, compared with those with an annual income of $15,000 and more, participants with an annual income between $5,000 and $9,999 had a lower likelihood of attrition (OR = 0.64, 95% CI = [0.422, 0.972]). Of the health and social well-being predictors, higher perceived loneliness and lower perceived social support were associated with a higher risk of attrition (OR = 1.14, 95% CI = [1.041, 1.242]; OR = 0.96, 95% CI = [0.930, 0.999], respectively).

**Table 4.** Binary logistic regression testing associations of baseline characteristics and retention status in those who were alive by Wave 5 in PINE (*N *= 2,541^a^).

Variables	Attrition (*N *= 655)	
	OR	95% CI
**Age**	1.01	(1.00, 1.03)
**Female**	1.02	(0.83, 1.25)
**Married**	1.06	(0.81, 1.37)
**Education**	1.02	(0.99, 1.04)
**Acculturation**	1.04***	(1.02, 1.06)
**Length of stay in the United States**	1.01**	(1.01, 1.02)
**Health insured**	0.70**	(0.55, 0.90)
**Annual income**		
** $0-$4,999**	0.82	(0.53, 1.26)
** $5,000-$9,999**	0.64*	(0.42, 0.97)
** $10,000-$14,999**	0.64	(0.40, 1.02)
** $15,000 and over (Ref.)**		
**Overall health status**		
** Very good/Good (Ref.)**		
** Fair**	1.00	(0.82, 1.24)
** Poor**	0.98	(0.72, 1.33)
**Medical comorbidities**	0.97	(0.90, 1.04)
**Depressive symptoms**	0.97	(0.95, 1.00)
**Loneliness**	1.14**	(1.04, 1.24)
**Cognitive function**	0.92	(0.78, 1.09)
**Perceived social support**	0.96*	(0.93, 1.00)
**Social strain**	0.97	(0.89, 1.04)
**Social engagement**	0.99	(0.98, 1.00)

*Note*. OR = odds ratio. CI = confidence interval.

a Analytic sample used complete cases, excluding those having missing data on independent variables.

* *p* < .05.

** *p* < .01.

*** *p* < .001.

We further conducted multinomial logistic regression to examine the effect of participant characteristics on attrition patterns (i.e., retention [reference group] *vs* early drop-out *vs* late drop-out *vs* drop-in). Results showed that older age was associated with a lower likelihood of drop-in (OR = 0.98, 95% CI = [0.953, 0.999]) but a higher likelihood of late drop-out (OR = 1.05, 95% CI = [1.023, 1.087]). Also, higher educational attainment was associated with an increased likelihood of early drop-out (OR = 1.05, 95% CI = [1.008, 1.083]). Higher acculturation was associated with an increased likelihood of all attrition groups (see Table 5). Longer length of stay in the US was associated with a higher likelihood of early and late drop-outs (OR = 1.01, 95% CI = [1.002, 1.027]; OR = 1.02, 95% CI = [1.004, 1.038]; respectively), but not drop-in (OR = 1.01, 95% CI = [0.997, 1.023]). Having health insurance was associated with a decreased likelihood of early drop-out (OR = 0.49, 95% CI = [0.345, 0.690]). Compared with those with an annual income of $15,000 and more, participants who had an annual income between $10,000 and $14,999 had a lower likelihood of late drop-out (OR = 0.27, 95% CI = [0.086, 0.836]). In addition, loneliness was associated with an elevated likelihood of early drop-out (OR = 1.20, 95% CI = [1.071, 1.353]).

**Table 5.** Multinomial logistic regression testing associations of baseline characteristics and attrition patterns (*N *= 2,541^a^).

Variables	Early drop-out (*n *= 276)	Late drop-out (*n *= 125)	Drop-in (*n *= 254)
	OR	OR	OR
**Age**	1.02	1.05***	0.98*
**Female**	1.22	1.02	0.85
**Married**	1.32	0.88	0.93
**Education**	1.05*	1.02	0.99
**Acculturation**	1.05***	1.04*	1.04*
**Length of stay in the United States**	1.01*	1.02*	1.01
**Health insured**	0.49***	0.83	0.99
**Annual income**			
** $0–$4,999**	0.92	1.06	0.65
** $5,000–$9,999**	0.71	0.66	0.60
** $10,000–$14,999**	0.65	0.27**	0.78
** $15,000 and over (Ref.)**			
**Overall health status**			
** Very good/Good (Ref.)**			
** Fair**	1.01	1.40	0.84
** Poor**	1.09	0.83	0.95
**Medical comorbidities**	0.95	0.96	0.99
**Depressive symptoms**	0.97	1.01	0.95
**Loneliness**	1.20**	1.03	1.12
**Cognitive function**	0.86	1.32	0.83
**Perceived social support**	0.96	0.98	0.96
**Social strain**	0.93	1.03	0.97
**Social engagement**	0.99	0.98	1.00

*Note.* OR = odds ratio. The retention group is the reference group.

a Analytic sample used complete cases, excluding those having missing data on independent variables.

* *p* < .05.

** *p* < .01.

*** *p* < .001.

## Discussion

To our knowledge, this is the first study to examine participant attrition in aging cohort studies among older Asian immigrants in the US. Several findings emerged from this study. First, this study indicated various attrition patterns in a large longitudinal study of older Chinese immigrants. Second, regression analyses showed that participants with higher acculturation and longer length of stay in the US were more likely to drop out of the study. Also, participants with higher educational attainment and loneliness were more likely to be early drop-outs. In addition, participants of a younger age were more likely to be drop-ins but less likely to be late drop-outs. Finally, those with health insurance or having a lower annual income were less likely to drop out of the PINE study.

One of the most important findings from this study is the effect of immigration-related factors on participation retention. Our results suggest that older Chinese immigrants with higher acculturation and longer length of stay in the US are less likely to remain in the study. Our reported relationship between length of stay and attrition aligns with findings from previous studies showing that longer length of stay can be a barrier to research participation among older immigrant adults (Brugge et al., 2005; Li et al., 2016). However, the adverse effect of acculturation on retention appears inconsistent with prior research that suggests high acculturation as a facilitator to participation recruitment (Li et al., 2016). As higher acculturation is often associated with higher educational attainment in this population (Dong et al., 2015), our findings also appear to be inconsistent with previous studies indicating higher educational attainment as a protective factor for attrition (Cacioppo & Cacioppo, 2018; Heid et al., 2021). Indeed, we found that participants with higher educational attainment were more likely to be early drop-outs. There were several possible explanations for a higher likelihood of attrition among older Chinese immigrants with higher acculturation and educational attainment. First, participants with higher acculturation might have busier schedules, including work or social commitments, making it harder to prioritize research participation. Second, more acculturated individuals are more likely to live outside ethnic enclaves or relocate frequently due to better socioeconomic status, increasing the risk of losing contact. These findings demonstrate how immigration-related factors and socioeconomic status uniquely influence participation retention among older Chinese immigrants, highlighting the critical need for tailored engagement strategies—particularly for individuals with higher acculturation and educational attainment.

Another important finding of this study is the effect of perceived loneliness on participation retention. Our results showed that individuals with higher levels of perceived loneliness were more likely to be early drop-outs. This finding is in line with previous studies highlighting the challenges of engaging socially isolated individuals in research (Dare et al., 2019). This finding suggests the limitations in traditional engagement and retention efforts (e.g., monetary compensation, mail-based outreach) in retaining lonely older immigrant adults in longitudinal aging studies. Also, we found that perceived social support was associated with a lower likelihood of attrition, indicating that perceiving support from others might enhance retention. In addition, we did not find the effect of social engagement on attrition, despite prior research linking social engagement with other social well-being predictors, such as loneliness (McHugh Power et al., 2019). One possible explanation is a relatively weak correlation between social engagement and loneliness in the PINE study (data not shown), which may indicate that these constructs capture distinct aspects of social well-being and thus might have different effects on attrition in this population.

Notably, we found that a younger age was associated with a higher likelihood of drop-in. This finding suggests that lower participation in early follow-ups may not reflect a lack of interest in study participation, but rather other barriers to successful contact—particularly among younger-old participants. Through continued re-engagement efforts by the PINE team (i.e., birthday cards, calendars, multiple contact methods), younger participants might be more likely to re-engage in the study. Interestingly, we did not observe an elevated risk of attrition associated with older age in the PINE study, which is inconsistent with existing studies indicating that older age is a risk factor for attrition (Cacioppo & Cacioppo, 2018). One possible explanation might be due to the ongoing outreach effort of the PINE team, including regular contact with the participants and canvassing, to enhance continued participation. Another possible explanation might be the limited mobility of older participants in this sample, reducing the risk of attrition due to relocation.

In addition, we found that those with lower income were less likely to drop out of the study, suggesting the possible effect of participation incentives on retaining participants. However, this interpretation should be cautious, given that most of the PINE participants had low income, and the participation incentives were modest (ranging from $15 to $60 across follow-ups). Also, we found that participants with health insurance were more likely to remain in the study. This finding appears to contradict our observed relationship between lower income and greater retention, as lower income is often associated with higher rates of uninsurance (Lee et al., 2021). One possible explanation is that many PINE participants were eligible for Medicare (i.e., a federal health insurance program for adults age 65 or older), which may have reduced variation in health insurance coverage by income. Having health insurance among older immigrants may reflect greater engagement with formal systems of care, which might possibly translate into willingness to engage in health research. However, this result should be interpreted with caution, as many PINE participants might be eligible for Medicare (e.g., 78.4% of the PINE participants age 65 and older). Unexpectedly, our study did not observe the impact of health factors on attrition, though previous studies have suggested an elevated risk of attrition associated with poor health (Kuh et al., 2016). This null result might be due to the fact that the PINE study only involved survey assessments, and that a face-to-face home interview for data collection may overcome potential participation barriers related to health conditions, such as mobility limitations (Shapiro et al., 2017).

There are several implications of this study for conducting population-based aging studies among immigrant populations in the US. First, the relatively high retention rate (i.e., 74.2%) in the PINE study between 2011 and 2020 demonstrates the possible success of sustaining an aging cohort among Asian American immigrants, especially when operationally allowing for nonmonotone attrition patterns. Among the PINE participants in the attrition group, 38.4% who missed an earlier follow-up completed at least one subsequent wave, suggesting the potential to enhance continued participation through re-engagement strategies. Second, the different attrition patterns observed in this study and the distinct significant predictors for these attrition patterns suggest the potential value of tailoring retention strategies to maximize the impact of retention effort in enhancing continued participation. For example, participants with higher educational attainment were more likely to drop out early, indicating a need for early re-engagement efforts targeting this subgroup, which often also have higher acculturation, another risk factor for early drop-out observed in this study. Also, our results showed that individuals with lower income, overall, were less likely to drop out of the study, possibly explained by the finding that those with higher annual income were more likely to be late drop-outs. This finding highlights the benefit of even modest monetary compensation in motivating research participation, particularly in older immigrants with low income. However, this result also points to the limitations of financial incentives in sustaining long-term engagement, particularly for those with higher income, emphasizing the need for additional non-monetary strategies to promote continued participation. Interestingly, the retention group was not different from the attrition group with respect to social engagement, which may present an opportunity for researchers to link with current sites of social engagement (e.g., libraries, senior centers) to increase research engagement between data collection points.

A few limitations should be considered when interpreting findings from this study. First, although this study examined a comprehensive set of predictors of attrition, it did not capture participants’ underlying motivations, perceived barriers, or contextual factors that may influence retention. This limitation stems from the post-hoc nature of the attrition analysis using an existing longitudinal study that was not originally designed to examine reasons for attrition and retention. Future research would benefit from incorporating qualitative approaches to collect data on factors related to research engagement to provide deeper insight into the individual, interpersonal, and contextual drivers of continued participation or drop-out. For example, future studies could incorporate open-ended questions during follow-up assessments to allow participants to share their reasons for continued participation. When feasible, brief exit interviews could be conducted with participants who choose to withdraw from the study to better understand their motivations for dropping out. Such practices can provide timely and actionable feedback to inform retention strategies aimed at sustaining participation and minimizing attrition. Second, the absence of data on the total number of participants retained through specific PINE retention strategies limited our ability to evaluate their effectiveness in reducing attrition. Also, the lack of detailed information on reasons for attrition prevented us from examining differences in drop-out by cause. Third, the PINE study’s exclusive reliance on in-home survey assessments limits the generalizability of its attrition findings to aging studies that incorporate multimodal data collection methods, such as clinical visits and biospecimen sampling. Finally, findings from this study may not be generalizable to older Chinese immigrants living in other areas or other racial or ethnic aging populations.

## Conclusion

This study is among the first studies to evaluate retention in aging studies among older immigrant populations. Results from this study indicate unique risk factors for attrition among older Chinese immigrants in the US, including high acculturation, high socioeconomic status, and elevated loneliness. These findings provide important insights into developing targeted retention strategies in longitudinal aging research with this population. Future studies should evaluate the effectiveness of retention strategies in reducing attrition, as addressing this issue is critical to minimizing potential attrition biases in aging research.

## Data Availability

The study materials and analytic code will be made available upon request to the corresponding author (Y. Jiang) at yanping.jiang@ifh.rutgers.edu. Data for this study is not publicly available, and the data access is managed by the Rutgers Institute for Health, Health Care Policy and Aging Research Survey/Data Core (Core). Rutgers and the Core maintain PINE datasets in a National Institutes of Health compliant core resource facility. Access to the source data held at Rutgers will be granted to investigators who obtain data use agreements from the Core.
